# Hand grip strength and fatigability: correlation with clinical parameters and diagnostic suitability in ME/CFS

**DOI:** 10.1186/s12967-021-02774-w

**Published:** 2021-04-19

**Authors:** Bianka Jäkel, Claudia Kedor, Patricia Grabowski, Kirsten Wittke, Silvia Thiel, Nadja Scherbakov, Wolfram Doehner, Carmen Scheibenbogen, Helma Freitag

**Affiliations:** 1 Institute of Medical Immunology, Charité - Universitätsmedizin Berlin, Corporate Member of Freie Universität Berlin, Humboldt Universität zu Berlin and Berlin Institute of Health, 13353 Berlin, Germany; 2 MVZ Onkologie Havelhöhe, Berlin, Germany; 3 Department of Cardiology (Virchow Klinikum), Charité - Universitätsmedizin Berlin, Corporate Member of Freie Universität Berlin, Humboldt Universität zu Berlin, and Berlin Institute of Health, 13353 Berlin, Germany; 4 Berlin Institute of Health Center for Regenerative Therapies (BCRT), Charité University Medicine Berlin, Berlin, Germany; 5 DZHK (German Centre for Cardiovascular Research), Partner Site Berlin, Berlin, Germany

**Keywords:** Myalgic encephalomyelitis/chronic fatigue syndrome, Diagnostic, Handgrip, Muscular fatigue, Muscle strength, Muscular recovery

## Abstract

**Background:**

Myalgic Encephalomyelitis/Chronic Fatigue Syndrome (ME/CFS) is a complex and debilitating disease accompanied by muscular fatigue and pain. A functional measure to assess muscle fatigability of ME/CFS patients is, however, not established in clinical routine. The aim of this study is to evaluate by assessing repeat maximum handgrip strength (HGS), muscle fatigability as a diagnostic tool and its correlation with clinical parameters.

**Methods:**

We assessed the HGS of 105 patients with ME/CFS, 18 patients with Cancer related fatigue (CRF) and 66 healthy controls (HC) using an electric dynamometer assessing maximal (Fmax) and mean force (Fmean) of ten repetitive measurements. Results were correlated with clinical parameters, creatinine kinase (CK) and lactate dehydrogenase (LDH). Further, maximum isometric quadriceps strength measurement was conducted in eight ME/CFS patients and eight HC.

**Results:**

ME/CFS patients have a significantly lower Fmax and Fmean HGS compared to HC (p < 0.0001). Further, Fatigue Ratio assessing decline in strength during repeat maximal HGS measurement (Fmax/Fmean) was higher (p ≤ 0.0012). The Recovery Ratio after an identical second testing 60 min later was significantly lower in ME/CFS compared to HC (Fmean2/Fmean1; p ≤ 0.0020). Lower HGS parameters correlated with severity of disease, post-exertional malaise and muscle pain and with higher CK and LDH levels after exertion.

**Conclusion:**

Repeat HGS assessment is a sensitive diagnostic test to assess muscular fatigue and fatigability and an objective measure to assess disease severity in ME/CFS.

**Supplementary Information:**

The online version contains supplementary material available at 10.1186/s12967-021-02774-w.

## Background

Chronic Fatigue Syndrome (also known as Myalgic Encephalomyelitis, ME/CFS), is a complex disease with persistent mental and physical fatigue causing severe impairment of quality of life. The cardinal symptom is exertional intolerance with post-exertional malaise (PEM), which describes a disproportionate intensification of symptoms and a prolonged regeneration phase after physical or mental effort [1–3]. Muscle fatigability is another important hallmark in which muscles become weaker after exertion and it can last for days before full muscle strength is restored.

To date, the etiology and pathophysiology of ME/CFS is still unresolved, but there is ample evidence for a disturbed vascular regulation [4]. Diagnosing of ME/CFS is challenging for patients and physicians due to many unspecific symptoms, the broad differential diagnosis of chronic fatigue and the lack of an established biomarker. Currently, ME/CFS is a clinical diagnosis using comprehensive clinical evaluation and diagnostic criteria with the international Canadian Consensus Criteria (CCC) formulated by Carruthers et al*.* in 2003 as the most accepted [5]. However, to date ME/CFS patients are often un- or misdiagnosed as depression or burn-out, leading probably to a marked underestimation of prevalence [1, 6].

As objective measure to assess exertion intolerance in ME/CFS a repeat cardiopulmonary exercise test (CPET) is recommended by the National Institute of Health (NIH). Studies showed the maximum oxygen uptake and workload is significantly reduced at the day two of CPET [7]. However, the repeated exercise tests frequently lead to severe PEM and cannot be recommended for most patients [8].

Assessment of disease severity is mostly relied on questionnaires. Fatigue is a complex symptom and can be both mental and physical related to impaired muscle performance. Physical fatigue can be assessed objectively. Assessment of hand grip strength (HGS) is an established and highly reproducible tool to assess muscular strength and provides information about the person's physical function and state of health [9]. First studies showed that HGS is impaired in ME/CFS [10–12]. A recent study showed that maximal handgrip strength was significantly correlated with peak oxygen uptake and can predict maximal physical performance in CPET [13]. In the study by Meeus et al*.* also the HGS recovery after 45 min was analyzed and found to be impaired, while it was normal in HC [10]. Another approach to assess muscular force is the measurement of the quadriceps strength (QS). In an earlier study, ME/CFS patients showed lower QS and a prolonged recovery compared to the control group when performing consecutive contractions [14], another study reported normal quadriceps muscle force in ME/CFS [15].

Chronic fatigue occurs in many other diseases including cancer. About 30% of cancer survivors suffer from cancer-related fatigue (CRF) years after treatment severely impairing patients’ quality of life [16]. In order to assess specificity of HGS assessment for ME/CFS we included a cohort of CRF patients in our study.

In this study we evaluate repeat HGS measurement for assessing muscle fatigue in ME/CFS. By performing repeat hand grip testing fatigability and recovery of muscle strength could be assessed as diagnostic test.

## Methods

### Patients and controls

105 patients with ME/CFS and 18 patients with CRF who presented at the outpatient clinic for fatigue at the Institute for Medical Immunology at the Charité Berlin from December 2018 to January 2021 were included in the study. ME/CFS patients were diagnosed based on the Canadian Consensus Criteria (CCC) and exclusion of other medical or neurological diseases which may cause fatigue [5]. Further inclusion criteria were age ≤ 18 years or participation in an interventional study. CRF patients were diagnosed according to Cella criteria [16] and had to be in complete remission for at least six months. 66 age and sex–matched HC with self-reported healthy status served as control group. For QS measurement we included eight female ME/CFS patients and eight female healthy controls.

### Hand grip measurement

We measured the HGS with a digital hand dynamometer (CAMRY, model: SCACAM-EH101) in two separate sessions with a recovery break of 60 min between the sessions. The participant had to sit in an upright position and place the forearm of the dominant hand on a standard table in full supination. Before the start of the measurement, all participants had the opportunity to pull the handle twice to become familiar with the device. The handle was pulled with maximum force for three seconds followed by a five second relaxation phase under supervision by the study nurse. Within one session this procedure was repeated ten times with the dominant hand. After 60 min without any strenuous physical activity, a second session was conducted. Participants were verbally motivated during the measurement to continue using their maximum strength and to perform all repetitions. The dynamometer measures the highest value reached within the three seconds (force measurement in kg). The attempt with the highest measurement out of the ten repetitions was recorded as maximum strength.

### Assessment of Fmax and Fmean strength, fatigability and recovery

To estimate strength, fatigability and recovery we examined several parameters and ratios listed in Table 1.

**Table 1:** Parameters of muscle strength assessed by handgrip measurements

Parameter/Ratio	Formula	Explanation
Fmax	Fmax [kg]	Maximum grip strength within one session (ten repeat trials)
Fmean	$\frac{\sum 10\mathrm{ pulls }}{10}$	Mean grip strength of all ten trials
Fatigue ratio (assessment of fatigability)	$\frac{\mathrm{Fmax}}{\mathrm{Fmean}}$	Higher values indicate stronger decrease of force during one session
Recovery ratio (assessment of recoverability)	$\frac{\mathrm{Fmean}2}{\mathrm{ Fmean}1}$	Low values indicate impaired recovery

### Quadriceps strength measurement

Maximal isometric muscle strength of the quadriceps muscle (expressed in Newton, N) was measured as described in previous publications [17]. Briefly, the freely hanging leg of the sitting participant was connected at the ankle with a pressure transducer (Multitrace 2, Lectromed, Jersey, Channel Islands). Baseline maximal isometric strength was assessed from the best of three contractions on each leg, with a resting period of at least 60 s in between.

Afterwards the quadriceps muscle fatigue protocol was performed on the stronger leg as described previously [18]. In brief, participant performed repeated contractions at 30–40% of the maximum strength for one second, followed by one second of relaxations, using an acoustic signal as a guide. This cycle was performed for 40 s, followed by 20 s of rest. The 60 s cycle was repeated for 20 min. Participants were asked to undertake a maximal contraction at 5, 10, 15 and 20 min.

### Assessment of symptoms by scores

Each participating ME/CFS patient filled in the following questionnaires.

#### Bell score

Assessment of disease severity and everyday restrictions ranging from zero (total loss of self-dependence) to 100 (without restrictions) [19].

#### SF-36—Health status questionnaire

Assessment of physical function ranging from zero (greatest possible health restrictions) to 100 (no health restrictions) [20].

#### Chalder Fatigue Scale

Assessment of the severity of fatigue on a scale from zero (no fatigue) to 33 (heavy fatigue) [21].

#### COMPASS-31

Assessment of autonomic dysfunction including vasomotor, orthostatic, ocular, bladder and gastrointestinal symptoms ranging from zero (without symptoms) to 100 (strong autonomic dysfunction) [22] as was shown previously in ME/CFS patients [23].

#### PEM criteria

Assessment of frequency, severity and duration of post-exertional malaise (PEM) symptoms, such as muscle weakness, pain or mental tiredness, ranging from 0 (no PEM) to 46 (frequent, severe and long PEM) [24].

#### Symptom Score

Quantification of symptoms of the Canadian Consensus Criteria (one = no symptoms to ten = extreme symptoms) [25, 26].

### Assessment of CK and LDH

Creatine kinase (CK, immunological UV-test) and lactate dehydrogenase (LDH, photometric test) were determined from blood samples drawn after the second HGS assessment at the Charité diagnostics laboratory (Labor Berlin GmbH, Berlin, Germany).

### Statistical analysis

We performed the statistical analysis with the software GraphPad Prism 6.0. To exclude physiological, sex-related differences in strength, we studied male and female subjects separately. Gaussian distribution of data was examined by D’Agostino and Pearson test. Accordingly, we used unpaired t-tests and Mann–Whitney test to analyze differences between the groups. For comparison of the first and the second session we used paired t-test. For detection of linear and non-linear correlations we calculated Pearson and Spearman correlation coefficients, respectively. For analyzation of the diagnostic ability of handgrip measurements and determination of discrimination thresholds between HC and ME/CFS, we performed receiver operator characteristics (ROC) analyses and computed sensitivity and specificity. A p-value of < 0.05 was considered as statistically significant. Due to multiple testing p-values are considered descriptive.

## Results

### Study population

105 ME/CFS patients were enrolled for HGS measurement. Control groups were 66 age- and sex-matched healthy controls (HC) and 18 female patients with cancer related fatigue (CRF). Table 2 provides further characteristics of the participants.

**Table 2:** Characteristics of the study population

	HC		ME/CFS		CRF
Sample size (n)	66		105		18
	Female	Male	Female	Male	Female
	36	30	61	44	18
Age (years)	42 (22–62)	33.5 (21–62)	49 (21–76)	40 (18–60)	44.5 (19–63)
Bell Score	–	–	30 (10–65)	40 (20–70)	35 (30–80)
Duration of disease (years)	–	–	4 (1–32)	4 (1–42)	6 (1–16)
Post-infectious ME/CFS	–	–	n = 52	n = 40	–

Median values with range in brackets

*HC* healthy controls, *ME/CFS* myalgic encephalomyelitis/chronic fatigue syndrome, *CRF* cancer related fatigue, *Bell* Bell Score

### Fmax and Fmean HGS

**Fig. 1:**
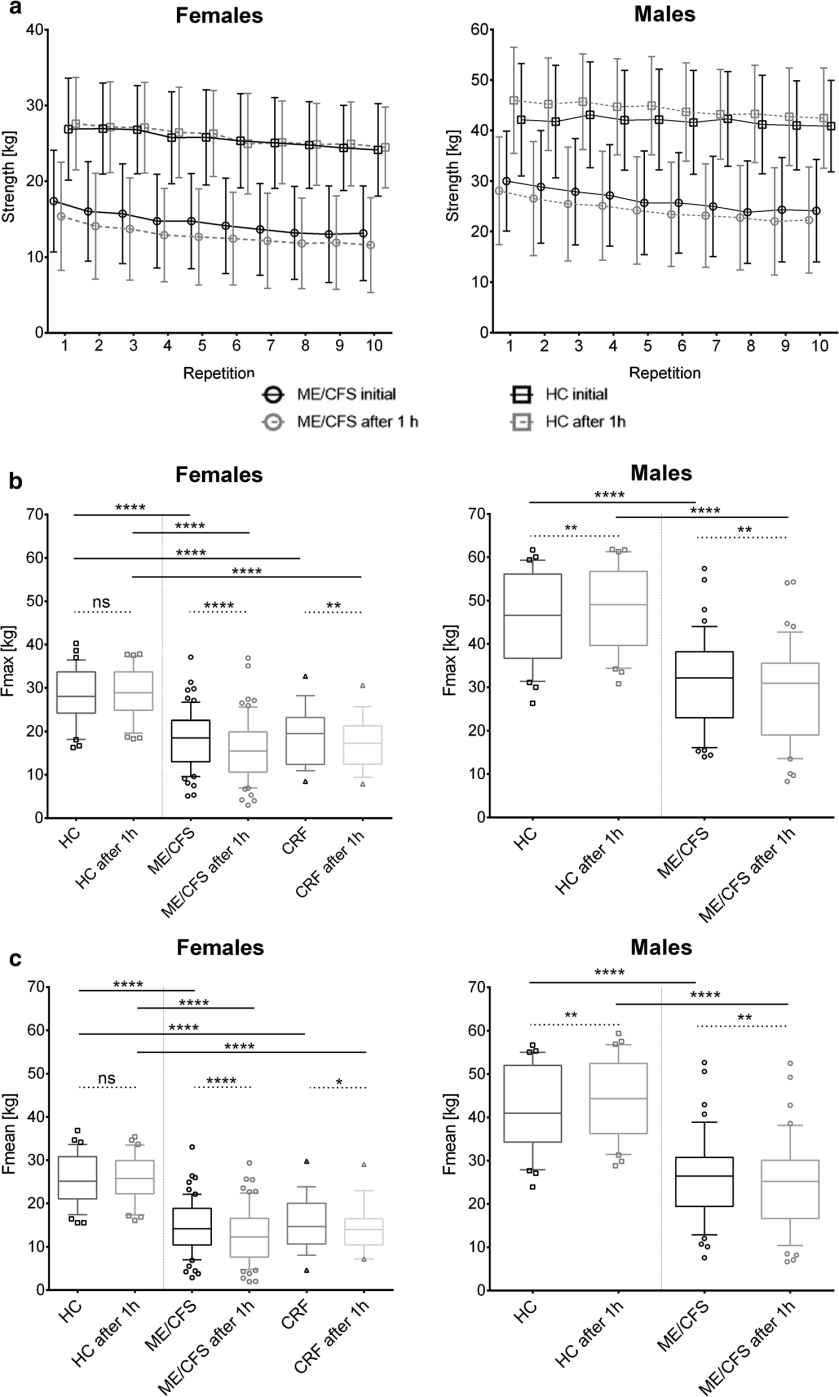
Handgrip strength (**a**), Fmax (**b**) and Fmean (**c**). ME/CFS patients showed lower levels and stronger decrease of HGS compared to HC over ten repeat pulls resulting in significantly diminished Fmax and Fmean in the patient group. Further, Fmax and Fmean dropped significantly after one hour in ME/CFS as well as in CRF patients, whereas it remains unchanged in healthy women or even raised in healthy men. Ten repeat measurements of maximal HGS were performed in two sessions (60-mininterval) by a hand dynamometer (in kg). Left: female ME/CFS patients (circles, n = 61), CFR patients (triangles, n = 18) and HC (squares, n = 36); right: male ME/CFS patients (circles, n = 44) and (HC, (squares, n = 30). Continuous line: initial session, dotted line: second session after one hour. Boxplots 10–90 percentile with outliers, ns = p > 0.05, *p < 0.05, **p < 0.01, ***p < 0.001, ****p < 0.0001 (Mann–Whitney-Test for comparison between groups, paired-t-test for comparison of both sessions). Mean values in kg with SD

All 189 participants completed ten consecutive maximal HGS measurements which were repeated after 60 min. Both male and female ME/CFS patients showed strongly reduced maximal and mean HGS in the first session (Fmax1, Fmean1) and the second session after one hour (Fmax2, Fmean2) compared to HC (Table 3 and Fig. 1, all p < 0.0001). In female CRF patients Fmax and Fmean in both sessions were also significantly diminished compared to female HC (Table 3, Fig. 1, both p < 0.0001).

**Table 3:** Results of handgrip strength measurement

	Females						Males			
	HC, n = 36		ME/CFS, n = 61		CRF, n = 18		HC, n = 30		ME/CFS, n = 44	
Fmax1	28.2 (6.25)		18.1 (6.68)	*p* < *0.0001*	18.9 (6.73)	*p* < *0.0001* *p* < *0.0001*	45.8 (10.2)		31.2 (10.4)	*p* < *0.0001*
Fmax2	28.7 (5.73)		16.0 (7.14)	*p* < *0.0001*	17.2 (6.00)		48.3 (9.58)		29.2 (10.9)	*p* < *0.0001*
Fmean1	25.6 (5.87)		14.6 (6.09)	*p* < *0.0001*	15.5 (6.34)	*p* < *0.0001* *p* < *0.0001*	41.8 (9.66)		26.3 (9.91)	*p* < *0.0001*
Fmean2	25.9 (5.46)		12.9 (6.24)	*p* < *0.0001*	14.3 (5.74)		44.2 (9.32)		24.5 (10.4)	*p* < *0.0001*
Fatigue Ratio 1	1.105 (0.09)		1.300 (0.297)	*p* < *0.0001*	1.261 (0.21)	*p* = *0.0001*	1.097 (0.04)		1.213 (0.18)	*p* = *0.0006* *p* = *0.0012*
Fatigue Ratio 2	1.113 (0.07)		1.299 (0.25)	*p* < *0.0001*	1.223 (0.13)	*p* = *0.0004*	1.097 (0.05)		1.216 (0.17)	
Recovery Ratio	1.0 (0.077)		0.87 (0.19)	*p* < *0.0001*	0.96 (0.26)	*p* = *0.0020*	1.1 (0.14)		0.92 (0.14)	*p* < *0.0001*
*Comparison of first and second measurement*										
Fmax1	28.2	*p* = 0.3295	18.1	*p* < *0.0001*	18.9	*p* = *0.0053*	45.8	*p* = *0.0048*	32.2	*p* = *0.0019*
Fmax2	28.7		16.0		17.2		48.3		29.2	
Fmean1	25.6	*p* = *0.3537*	14.6	*p* < *0.0001*	15.5	*p* = *0.0376*	41.8	*p* = *0.0057*	26.3	*p* = *0.0022*
Fmean2	25.9		12.9		14.3		44.2		24.5	

Mean value of handgrip strength in kg and ratios with standard deviation in brackets; p-value refers to comparison with healthy controls (Mann–Whitney-Test) or, in the second compartment of the table, to comparison of both sessions (Paired t-test), respectively

Further, ME/CFS patients had a significantly lower Fmax and Fmean HGS in the second session compared to the first (Table 3 and Fig. 1). In female CRF patients, Fmax and Fmean were significantly reduced in the second session. In contrast female HC showed a similar Fmax and Fmean after one-hour break and male HC even a significant increase in Fmax and Fmean.

### Fatigability and recovery of HGS

**Fig. 2:**
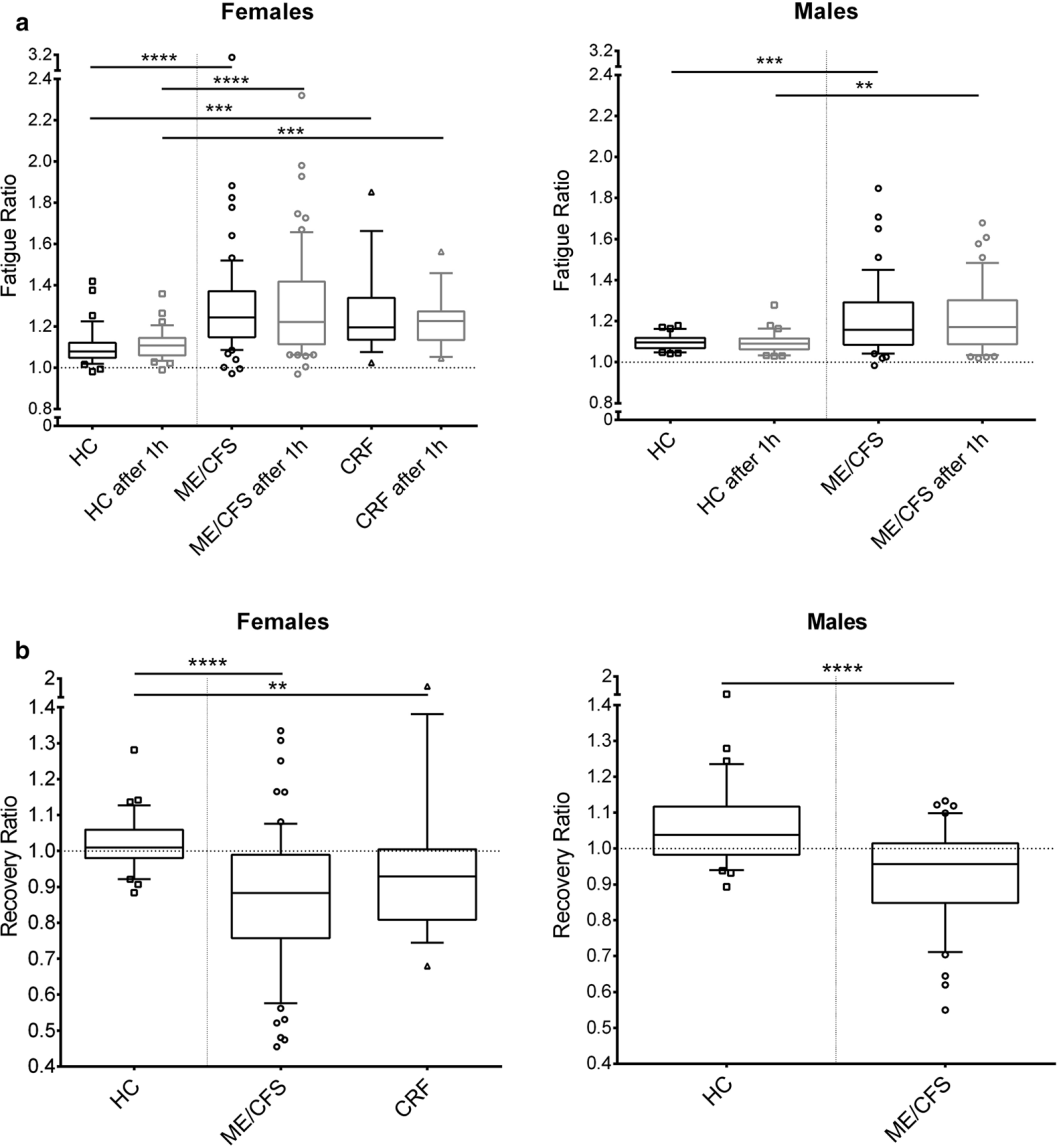
Fatigue ratio (**a**) and Recovery ratio (**b**) ME/CFS patients as well as female CRF patients showed higher Fatigue Ratios in both sessions and lower Recovery Ratios compared to HC. Ten repeat measurements of maximal HGS were performed in two sessions (60-min interval) by a hand dynamometer (in kg). Fatigue Ratio = (Fmax/Fmean), Recovery Ratio = (Fmean2/Fmean1). Left: female ME/CFS patients (circles, n = 61), CRF patients (triangles, n = 18) and HC (squares, n = 36), right: male ME/CFS patients (circles, n = 44) and HC (squares, n = 30). Boxplots (10–90 Percentile), ns = p > 0.05, *p < 0.05, **p < 0.01, ***p < 0.001, ****p < 0.0001 (Mann–Whitney-Test)

The Fatigue Ratio (Fmax/Fmean) was assessed as correlate of decrease of HGS during repeat measurements. ME/CFS patients had a stronger decrease in HGS resulting in higher Fatigue Ratios in comparison to HC in both first and second measurement (Table 3 and Fig. 2a). Further, Recovery Ratio of HGS (Fmean2/Fmean1) was diminished in the ME/CFS patients, as correlate of lower HGS during the second measurement, while HC had Recovery Ratios values of about one (Fig. 2b).

In female CRF patients, similar to ME/CFS, higher Fatigue Ratios 1 and 2 and reduced Recovery Ratios were observed compared to HC (Fig. 2 and Table 3).

### Correlation of HGS with symptom severity in ME/CFS patients

We next correlated HGS with parameters of symptom severity and disability (Table 4 and Additional file 1: Figures S1, Additional file 2: Figure S2 and Additional file 4: Table S1).

**Table 4:** Clinical characteristics of ME/CFS and correlations with handgrip strength

	Female			Male		
Bell	Median	Range	n	Median	Range	n
	30	10–65	59	40	20–70	42
Correlations	r	p	type	r	p	type
Fmax1	0.2670	**0.0409**	lin	0.115	0.4682	lin
Fmean1	0.1226	0.3551	non-lin	0.3255	**0.0354**	non-lin
Fmean2	0.1444	0.2710	non-lin	0.3175	**0.0405**	non-lin
Fatigue Ratio1	0.07946	0.5497	non-lin	− 0.3995	**0.0088**	non-lin
Fatigue Ratio2	− 0.211	0.0643	non-lin	− 0.3624	**0.0183**	lin

PEM	Median	Range	n	Median	Range	n
	37	9–46	35	34	21–46	35
Correlations	r	p	type	r	p	Type
Fmean2	− 0.334	**0.0499**	non-lin	− 0.1987	0.2526	non-lin
Fatigue Ratio2	0.4040	**0.0161**	lin	0.34	**0.0457**	lin

Muscle Pain	Median	Range	n	Median	Range	n
	8	0–10	50	6	1–10	39
Correlations	r	p	type	r	p	Type
Fmean2	− 0.3095	**0.0287**	lin	− 0.1643	0.3175	lin
Recovery Ratio	− 0.282	**0.0472**	non-lin	− 0.1542	0.3488	non-lin

COMPASS vasomotor	Median	Range	n	Median	Range	n
	0	0–5	49	0	0–5	40
Correlations	r	p	Type	r	p	Type
Fmax1	− 0.2664	0.0643	non-lin	− 0.3537	**0.0251**	non-lin
Fmax2	− 0.3635	**0.0102**	non-lin	− 0.4327	**0.0026**	non-lin
Fmean1	− 0.3013	**0.0354**	non-lin	− 0.3784	**0.0161**	non-lin
Fmean2	− 0.3663	**0.0096**	non-lin	− 0.5767	**< 0.0001**	non-lin
Fatigue Ratio2	**0.03817**	0.7946	lin	0.4869	**0.0014**	lin
Recovery Ratio	− 0.1938	0.1821	non-lin	− 0.628	**< 0.0001**	non-lin

SF-36 phys. funct	Median	Range	n	Median	Range	n
	40	5–90	52	47.5	0–100	38
Correlations	r	p	Type	r	p	Type
Fmax2	0.1724	0.2217	non-lin	0.3474	**0.0351**	non-lin
Fmean2	0.2326	0.0970	lin	0.3605	**0.0284**	lin

CK	Median	Range	n	Median	Range	n
	67.5	15–229	48	90	29–447	33
Correlations	r	p	Type	r	p	Type
Fatigue Ratio2	0.3054	**0.0348**	lin	− 0.1856	0.3092	lin
Recovery Ratio	− 0.2935	**0.0429**	lin	− 0.1045	0.5691	lin

LDH	Median	Range	n	Median	Range	n
	216	150–286	47	225.5	145–308	34
Correlations	r	p	Type	r	p	Type
Fatigue Ratio1	− 0.06696	0.6547	non-lin	0.3522	**0.0411**	non-lin
Fatigue Ratio2	0.3630	**0.0122**	non-lin	0.2683	0.125	non-lin
Recovery Ratio	− 0.0336	0.8226	non-lin	− 0.378	**0.0275**	non-lin

Clinical parameters with significant correlations with HGS and HGS ratios. Pearson (lin) or Spearman (non-lin) correlation coefficients were calculated. Bold: significant correlations with p < 0.05

Lower HGS parameters correlated with more disability (Bell Score), post-exertional malaise and muscle pain. Specifically, the Bell score correlated with Fmax1 in females and with Fmean and Fatigue Ratios in males. Higher PEM scores correlated with lower Fmean2 in females and higher Fatigue Ratio2 in both sexes and more muscle pain with lower Fmean2 and Recovery Ratio in females. Strikingly, the COMPASS score for vasomotor dysregulation assessing changes of skin color on hands/feet correlated with Fmax2 and Fmean1,2 in both males and females and in Fmax1, Fatigue Ratio2 and Recovery Ratio in males, too. Physical functioning assessed by SF-36 questionnaire correlated with Fmax2 and Fmean2 in males.

No correlations were found with total COMPASS score and severity of fatigue assessed by Chalder Fatigue Scale (Additional file 4: Table S1).

### Correlation of HGS with CK and LDH post exertion in ME/CFS patients

Finally, we correlated the muscle enzymes CK and the pyruvate to lactate catalyzing enzyme LDH assessed after exertion with HGS parameters (Table 4). Both CK and LDH are marker of muscle damage, too. In females, higher CK correlated with higher Fatigue Ratio2 and lower Recovery Ratio. Higher Lactate dehydrogenase (LDH) concentrations correlated in men with lower Recovery Ratio and higher Fatigue Ratio1 and in the female patients with higher Fatigue Ratio2.

### Sensitivity and specificity of HGS assessment

**Fig. 3:**
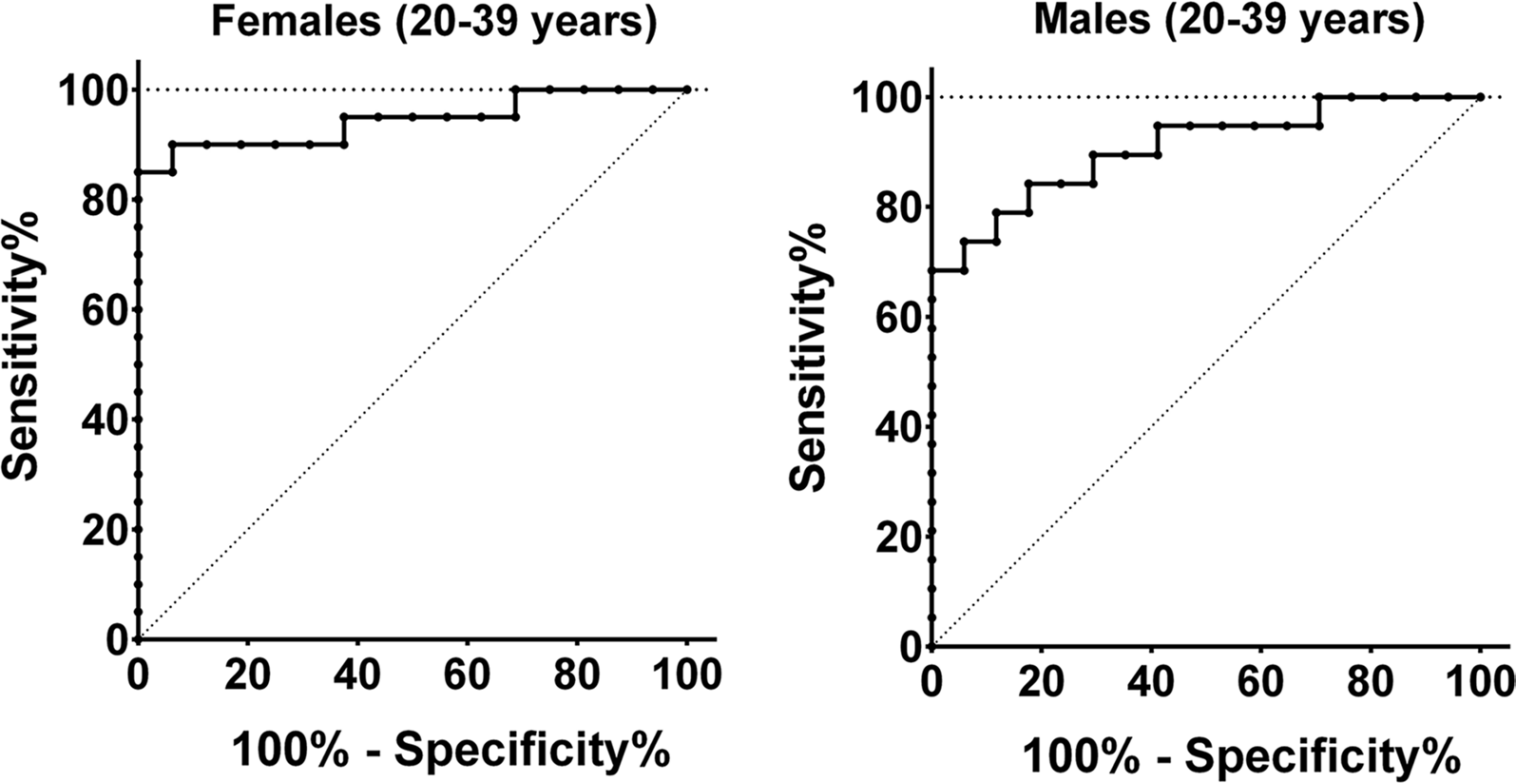
ROC analysis of Fmean2 Fmean2 reached a sensitivity of 85% and a specificity of 100% in female (left, ME/CFS: n = 20, HC:n = 16) and a sensitivity of 68% and a specificity of 100% in male (right, ME/CFS: n = 19; HC: n = 17) ME/CFS patients vs. HC aged 20–39 years

In order to analyze the suitability of HGS parameters as diagnostic test we conducted operator characteristics (ROC) analyses for the HGS parameters in males and females age-grouped in 20—39 years and 40 – 59 years. Cutoff values and area under the curve (AUC) for age 20–39 years are listed in Table 5. The highest values for sensitivity and specificity showed the Fmean2 with an AUC of 0.94 at a cutoff of < 19.95 kg for females and an AUC of 0.91 at a cutoff of < 28.76 kg for males (Fig. 3, Table 5 and Additional file 3: Figure S3). Cutoff values and AUC for females and males aged 40–59 years are shown in Additional file 5: Table S2.

**Table 5:** Cutoff values for hand grip strength (Age 20–39, ME/CFS vs HC)

	Sex	Cutoff Value	AUC	Sensitivity	Specificity
Fmax1	Females	< 23.55 kg	0.8656	70	93.75
	Males	< 33.35 kg	0.8762	68.42	94.12
Fmax2	Females	< 24.40 kg	0.9156	90	93.75
	Males	< 36.55 kg	0.9025	78.95	88.24
Fmean1	Females	< 19.74 kg	0.9188	80	100
	Males	< 29.36 kg	0.8947	73.68	94.12
Fmean2	Females	** < 19.95 kg**	**0.9438**	**85**	**100**
	Males	** < 28.76 kg**	**0.9071**	**68.42**	**100**
Fatigue Ratio1	Females	> 1.161	0.8500	75	87.5
	Males	> 1.197	0.5325	36.84	100
Fatigue Ratio2	Females	> 1.200	0.8563	65	93.75
	Males	> 1.189	0.5728	36.84	94.12
Recovery Ratio	Females	< 0.914	0.7906	70	93.75
	Males	< 1.125	0.5820	100	23.53

Results of ROC-Analysis for males and females aged 20–39 years. ME/CFS patients (females n = 20, males n = 19) and HC (females n = 16, males n = 17). Bold: most distinct parameter

### Quadriceps strength measurement

**Fig. 4:**
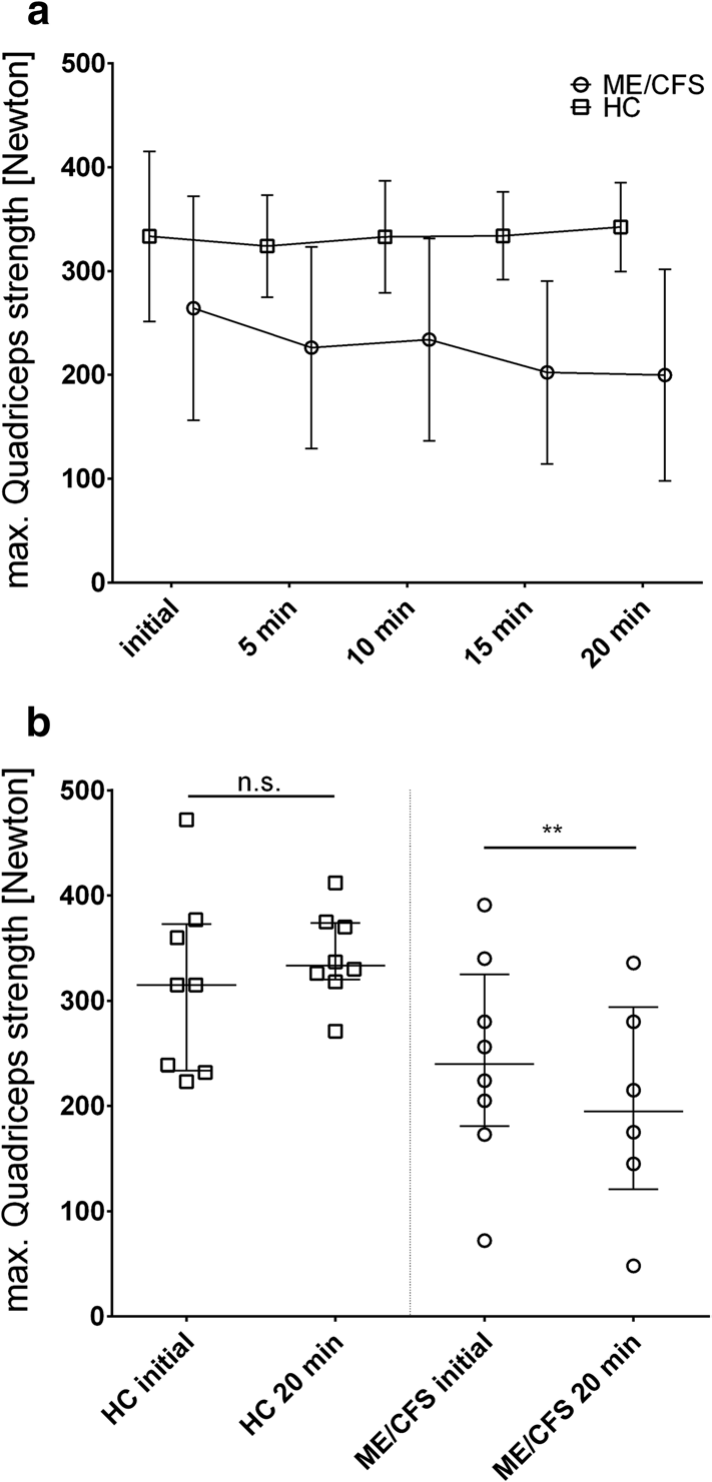
Quadriceps Strength over consecutive measurements (**a**) and Max strength (**b**) Female ME/CFS patients showed lower levels and stronger decrease of QS compared to HC upon repeat pulls (**a**) resulting in significantly diminished QS after 20 min (Fmax5) only in the patient group (**b**). **a** Mean value with standard deviation and **b** median with interquartile range, paired t-test. Squares: HC, n = 8, p = 0.7318, and circles: ME/CFS patients, n = 8, p = 0.0018

We performed five consecutive quadriceps strength (QS) measurements in eight female ME/CFS patients and eight female HC. Concordant to HGS, ME/CFS patients showed a significantly lower Fmax5 compared to Fmax1 compared to the HC resulting in a significantly lower Fmean (Table 6 and Fig. 4).

**Table 6:** Results of quadriceps strength measurement

Max. quadriceps strength [Newton]			
	HC, n = 8	ME/CFS, n = 8	
QS initial (Fmax1)	333 (82)	264 (108)	
5 min (Fmax2)	324 (46)	237 (88)	
10 min (Fmax3)	333 (50)	246 (88)	
15 min (Fmax4)	334 (39)	212 (85)	
20 min (Fmax5)	342 (40)	212 (90)	
Fmax5 in % of Fmax1	106.4 (21.8)	76.1 (13.0)	*p* = *0.0045*
Fmean	330 (46)	223.5 (88)	*p* = *0.013*
Fatigue Ratio	1.12 (0.058)	1.23 (0.110)	*p* = *0.1171*

Mean value of quadriceps strength with standard deviation in brackets; p-value refers to comparison with healthy controls (t-Test)

## Discussion

In our study we show that patients with ME/CFS have reduced Fmax and Fmean HGS compared to HC. Our finding of impaired Fmax is in line with previous studies [10–13]. In addition, the greater decrease of HGS during ten repeated measurements compared to HC resulted in a higher Fatigue Ratio as was shown already in the study by Neu et al*.* [12]. Further, upon repeat measurement after an hour HGS was significantly lower in ME/CFS while it was fully recovered in HC indicating an impaired recovery rate in patients with ME/CFS as a parameter for fatigability. This finding is in accordance with the study by Meeus et al*.* describing an impaired recovery 45 min after 18 repeat maximal contractions [10].

Our study provides further evidence that repeat measurement of muscle strength via a hand dynamometer is a simple and useful method to objectively detect muscular fatigue and fatigability. ROC analyses revealed a high diagnostic sensitivity and specificity to distinguish between HC and ME/CFS with Fmean2 yielding best discrimination in women and men in both age groups. For assessing HGS as diagnostic marker in ME/CFS, we think it is important to use the Canadian Consensus Criteria requiring PEM. In contrast Ickmans et al*.* using the “Fukuda criteria” for diagnosis, which do not require PEM, observed significant differences in strength and recovery only for ME/CFS patients with comorbid fibromyalgia [27, 28].

Our findings are, however, not specific for ME/CFS as CRF patients showed significantly lower HGS parameters compared to HC, too. We found fatigability and impaired recovery also in female CRF patients. For HGS in patients with multiple sclerosis (MS) two studies delivered inconsistent findings. Nacul et al*.* showed diminished Fmax in MS patients in contrast to normal Fmax and recovery of HGS in the study by Meeus et al*.* [10, 11].

The quadriceps force measurement provides evidence that diminished muscular strength is found in leg muscles, too, showing in a similar pattern as the HGS that ME/CFS patients have a stronger drop in force upon repeated exertion. Thus, our study confirms the findings by Paul et al*.* [14] and is in contrast to the report by Gibson et al*.* of normal quadriceps muscle function in ME/CFS [15].

The correlations between HGS and clinical parameters underline the clinical relevance of HGS assessment in ME/CFS. Lower Bell scores (higher severity of disability) correlated with lower Fmax1 in women and lower Fmax and Fmean and higher Fatigue Ratio1 in men indicating a causal relation of muscular strength to severity of the disease. Remarkably, PEM correlated in women with lower Fmean2 and in women and men with higher Fatigue Ratio2, providing evidence that HGS is an objective marker to assess the severity of PEM. In line with this, severity of muscle pain correlated with lower Fmean2 and impaired recovery in the female cohort. A potential explanation is that more severe PEM is associated with more muscular fatigability and muscle pain. This might explain why pacing as a strategy to avoid PEM results in improvement of physical fatigue [29]. In the studies by Nacul et al*.* and Neu et al*.* fatigue assessed by a fatigue questionnaire correlated with Fmax HGS. In the male cohort we found correlations between the SF-36 physical functioning, and Fmax2 and Fmean2. In our study we could not find an association of HGS parameters with the Chalder Fatigue Scale, which is, however, a questionnaire focusing mostly on mental fatigue (Additional file 6: Table S3).

Serum LDH and CK are both marker of muscle metabolism and damage and known to rise after exertion. Interestingly LDH and CK, which we determined after repeat HGS measurements also correlated with HGS parameters. Higher LDH concentrations correlated with higher Fatigue Ratio1/2 (Fmax/Fmean) and thus fatigability in both sexes. LDH is an enzyme catalyzing the conversion of pyruvate to lactate and may indicate a preferential energy production via glycolysis rather than the more efficient oxidative phosphorylation. Diminished pyruvate dehydrogenase function is described in ME/CFS resulting in increased conversion of pyruvate to lactate [30]. Thus enhanced LDH may indicate diminished capacity of oxidative phosphorylation, which may well explain lower muscle strength during repeat exercise. Further we found that higher CK levels are associated with poorer Recovery Ratio and higher Fatigue Ratio2 in women. CK plays a central role in the energy supply of the muscle and muscular strain can increase the serum concentration of CK, although there are inter-individual differences [31]. A previous study found that CK concentrations are lower in patients with severe ME/CFS [32, 33]. This is not in contrast to our study as CK was analyzed after exertion in our study.

The pathomechanism of muscular fatigue and fatigability in ME/CFS is not fully elucidated yet. Histologic alterations with an increase in the more fatigue-prone, energetically expensive fast fibre type was described in muscle biopsies from ME/CFS patients, while contractile properties of muscle fibres were preserved [34]. Further metabolic alterations with altered expression of genes for mitochondrial and energy function were described [35]. Another study reported impaired glucose uptake and adenosine monophosphate activated protein kinase (AMPK) activity in cultured muscle cells from ME/CFS patients [36]. Fluge et al*.* showed that myoblasts grown in presence of serum from patients with severe ME/CFS showed increased mitochondrial respiration and excessive lactate secretion indicating impaired pyruvate dehydrogenase function [30]. Impaired oxygen supply to muscles upon exertion in ME/CFS was described, too. ME/CFS patients showed evidence of reduced hyperemic flow and reduced oxygen delivery [37]. In line with this hypoperfusion in cerebral vessels was recently shown [38]. A MRI study revealed that patients with ME/CFS have abnormalities in recovery of intramuscular pH following exercise which is related to autonomic dysfunction [39]. Paradox vasoconstriction due to ß2 adrenergic dysfunction resulting in muscle hypoperfusion is considered as an important pathomechanism in ME/CFS [4]. In an own study we observed disease severity to correlate with endothelial dysfunction in ME/CFS [40]. In line with this concept, we observed here a strong correlation between vasomotor dysregulation assessed by COMPASS questionnaire with Fmax and Fmean of repeat testing.

A limitation of our study is that HGS may be influenced by inactivity although much less than leg muscle strength [41]. Further HGS is associated with age. Thus, a control group of physically inactive HC closely matched by age would be best for defining normal values of HGS parameters.

## Conclusion

HGS measurement is a simple diagnostic tool to assess the severity of muscle fatigue in ME/CFS. Repeat HGS assessment further allows to objectively assess fatigability and impaired recovery. Advantages of HGS measurement are easy handling, low cost and the low risk of causing PEM. Thus, it can be implemented easily in both primary care and research as an objective outcome parameter in clinical studies and drug development.

## Supplementary Information

**Additional file 1.** **Figure S1** Correlations in male ME/CFS patients. All relevant correlations of HGS measurements with other clinical parameters with p<0.005(Pearson and Spearman correlation coefficients, respectively). Line shows linear regression in Pearson correlations.

**Additional file 2.** **Figure S2** Correlations in female ME/CFS patients. All relevant correlations of HGS measurements with other clinical parameters with *p* < 0.005 (Pearson and Spearman correlation coefficients, respectively). Line shows linear regression in Pearson correlations.

**Additional file 3.** **Figure S3** Fmean2 and cutoff value Distinguishing between 20 – 39-year-old ME/CFS patients and HC using cut off values (dotted line) of Fmean2 determined by ROC analysis. Left:females (circles: ME/CFS, n=20, squares: HC, n=16), right: males (circles: ME/CFS, n=20, squares: HC, n=17).

**Additional file 4.** **Table S1**. Correlations of clinical parameters with HGS and HGS ratios. Pearson (lin) or Spearman (non-lin) correlation coefficients were calculated. Bold: significant correlations with *p* < 0.05.

**Additional file 5.** **Table S2**. Results of ROC-Analysis for females and males aged 40-59 years. ME/CFS patients (females n=35, males n=21) and HC (females n=18, males n=10). Bold: most distinct parameter.

**Additional file 6.** **Table S3**. Raw Data.

## Data Availability

All data generated or analyzed during this study are included in this published article and its Additional files.

## Abbreviations

AMPK: Adenosine monophosphate activated protein kinase
ANOVA: Analysis of variance
AUC: Area under curve
BMI: Body mass index
CCC: Canadian consensus criteria
CDC: Center of disease control
CFS: Chronic fatigue syndrome
CK: Creatine kinase
CPET: Cardiopulmonary exercise testing
CRF: Cancer related fatigue
HC: Healthy controls
HGS: Handgrip strength
LDH: Lactic dehydrogenase
ME: Myalgic encephalomyelitis
MS: Multiple sclerosis
NIH: National Institutes of Health
PEM: Post-exertional malaise
QS: Quadriceps strength
ROC: Receiver operating characteristic
SD: Standard deviation
