# Supplementary figures and images for: Hand grip strength and fatigability: correlation with clinical parameters and diagnostic suitability in ME/CFS
Source: J Transl Med. 2021 Apr 19;19:159. doi: 10.1186/s12967-021-02774-w (PMC8056497; doi:10.1186/s12967-021-02774-w)

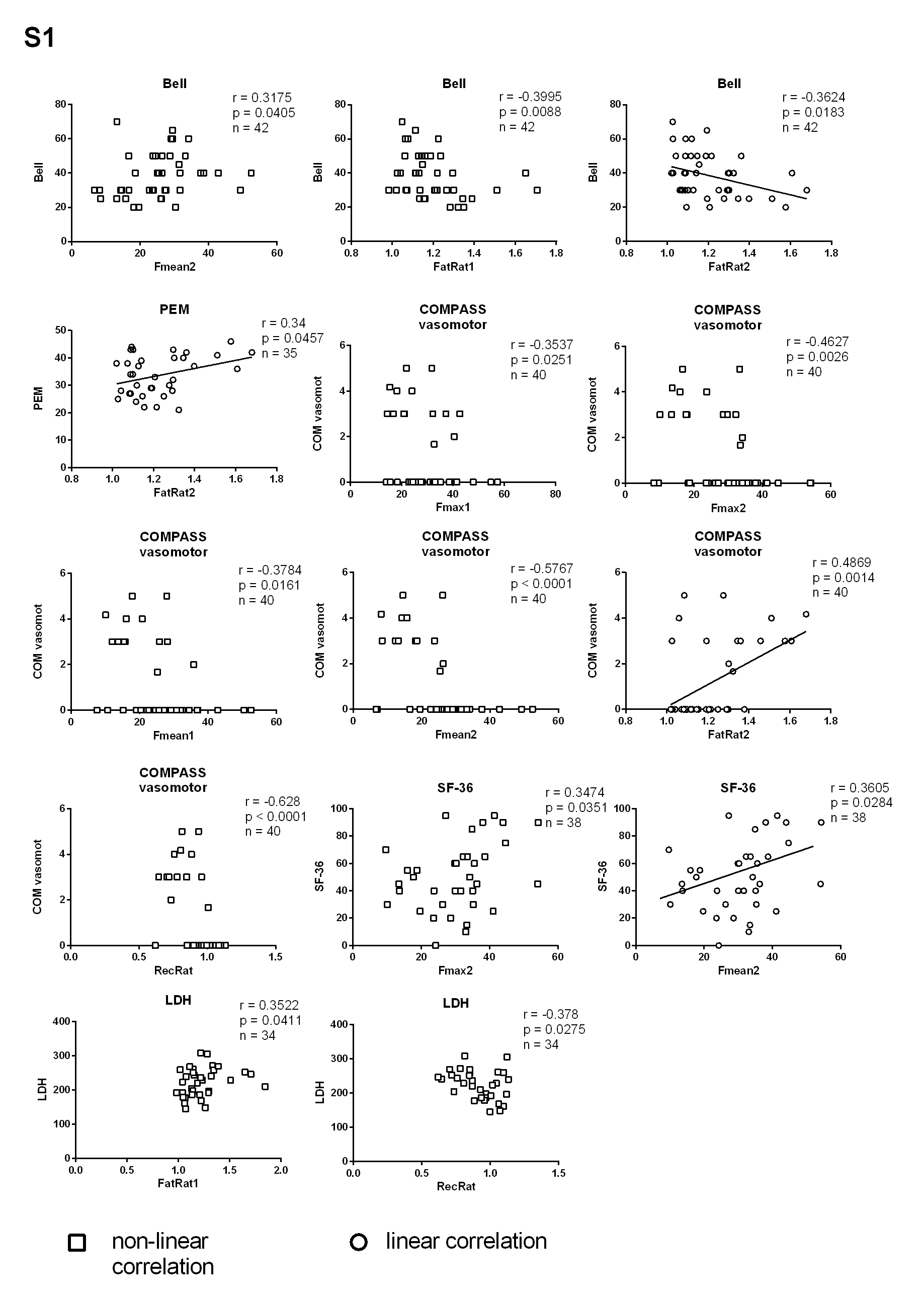

Supplement: Supplementary file 1 — Additional file 1. Figure S1 Correlations in male ME/CFS patients. All relevant correlations of HGS measurements with other clinical parameters with p<0.005(Pearson and Spearman correlation coefficients, respectively). Line shows linear regression in Pearson correlations. [file 12967_2021_2774_MOESM1_ESM.jpg]

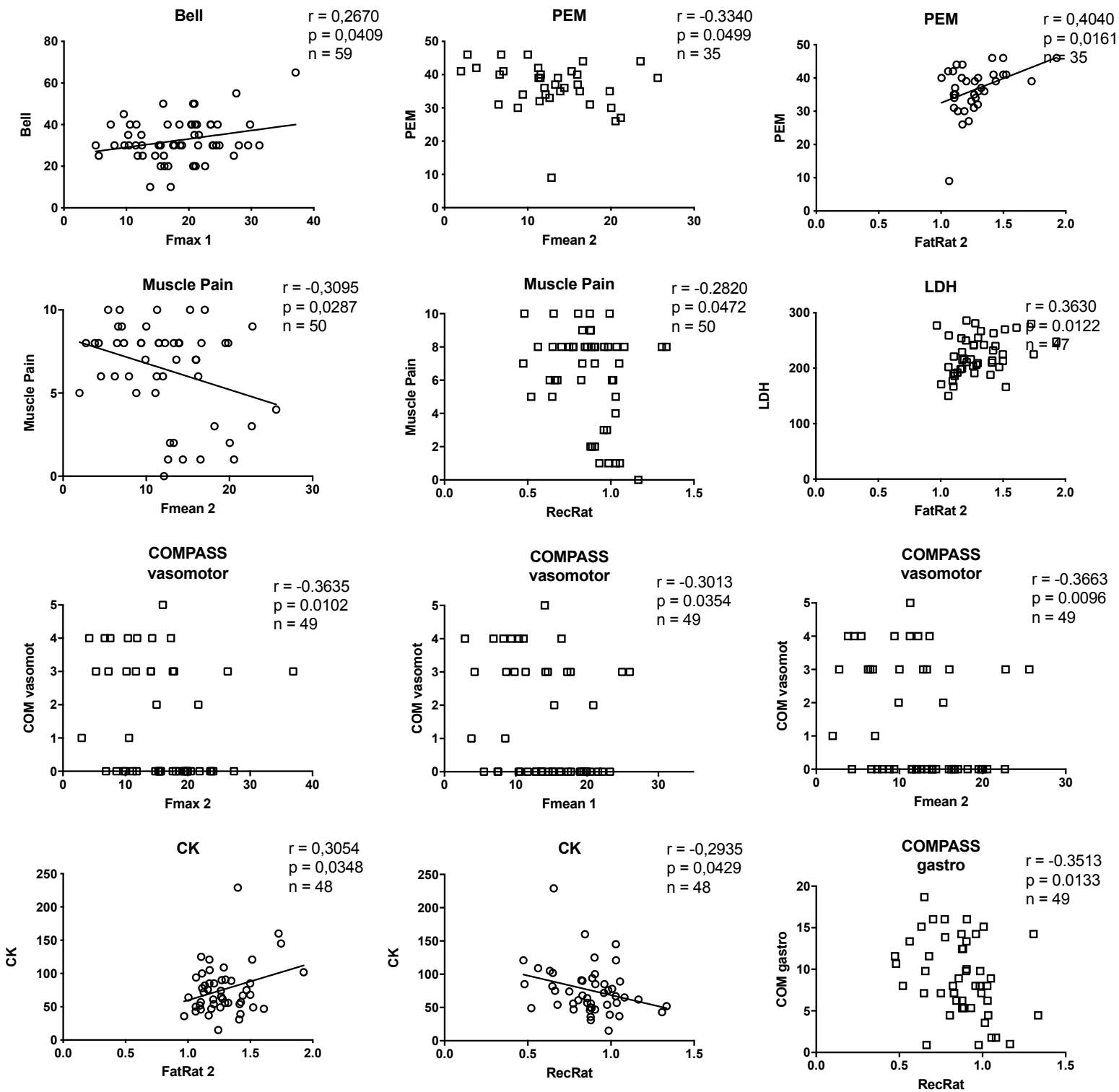

□ non-linear correlation

○ linear correlation

Supplement: Supplementary file 2 — Additional file 2. Figure S2 Correlations in female ME/CFS patients. All relevant correlations of HGS measurements with other clinical parameters with p < 0.005 (Pearson and Spearman correlation coefficients, respectively). Line shows linear regression in Pearson correlations. [file 12967_2021_2774_MOESM2_ESM.pdf]

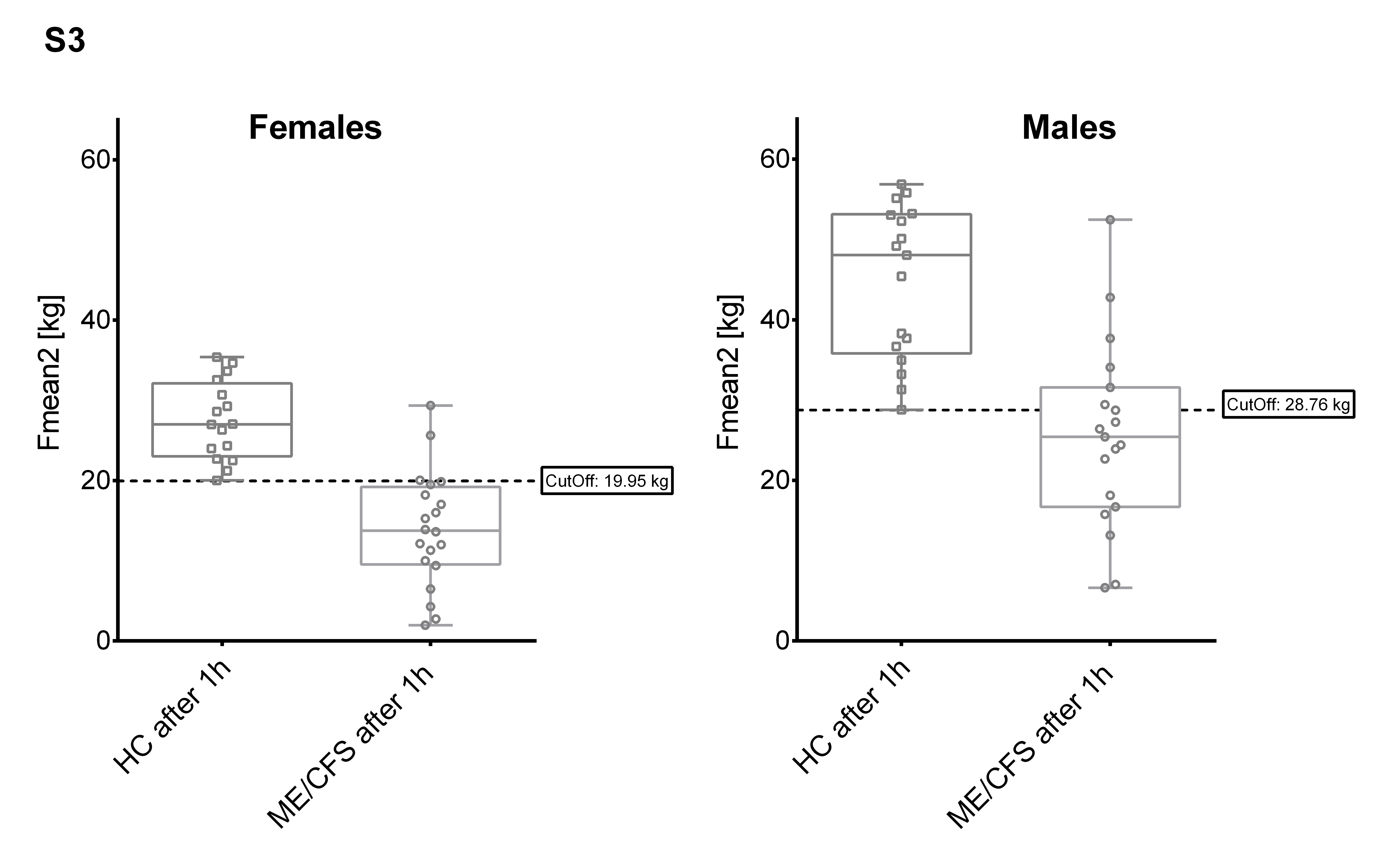

Supplement: Supplementary file 3 — Additional file 3. Figure S3 Fmean2 and cutoff value Distinguishing between 20 – 39-year-old ME/CFS patients and HC using cut off values (dotted line) of Fmean2 determined by ROC analysis. Left:females (circles: ME/CFS, n=20, squares: HC, n=16), right: males (circles: ME/CFS, n=20, squares: HC, n=17). [file 12967_2021_2774_MOESM3_ESM.jpg]
